# Evolution of Duplicated Hox Gene Clusters in Land Snails and Slugs

**DOI:** 10.1002/jez.b.23322

**Published:** 2025-08-06

**Authors:** Finn McHale, Peter O. Mulhair, Peter W. H. Holland

**Affiliations:** ^1^ Department of Biology University of Oxford Oxford UK; ^2^ Institute of Infection, Veterinary and Ecological Sciences University of Liverpool Liverpool UK

## Abstract

Terrestrial slugs and snails, order Stylommatophora, underwent a genome duplication in their ancestry. This affords an opportunity to examine how Hox gene clusters evolved after duplication in an invertebrate group and compare to the well‐studied genome duplications of vertebrates. Using genomic data and PCR verification, we describe Hox gene organization for 10 species of Stylommatophora and one close relative. All Stylommatophora sampled have two broken Hox gene clusters. The HoxA cluster is dispersed along one chromosome and generally has 9 genes, but only 8 genes in slugs. The HoxB cluster is dispersed along a different chromosome and usually has 7 genes, but only 6 genes in giant African land snails. No cluster has a full complement of 11 genes. The patchwork retention of duplicated Hox genes shows striking similarities to duplicated vertebrate Hox gene clusters.

## Graphical abstract

Molluscs of the order Stylommatophora underwent an ancient genome duplication. We show that stylommatophoran snails and slugs have two broken and incomplete Hox gene clusters; HoxA generally has 9 genes, HoxB usually has 7 genes. After duplication of an ancestral 11‐gene Hox cluster, there was patchwork retention of duplicated genes.

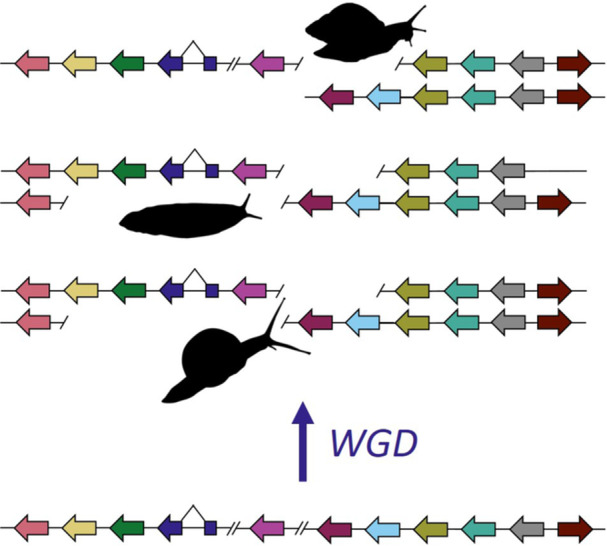

## 1 Introduction

Whole genome duplications seem to have been rarer during the diversification of animals than during the evolution of plants. In animals, our best understanding of the evolutionary impact of genome duplication comes from vertebrates. There was a genome duplication event in early vertebrate ancestry, followed by additional genome duplications in jawed and jawless vertebrates (Lundin 1993; Holland et al. 1994; Putnam et al. 2008; Simakov et al. 2020; Marlétaz et al. 2024; Yu et al. 2024), another genome duplication in the stem lineage of teleost fish (Taylor et al. 2003), and a separate event in the ancestor of sturgeon and paddlefish (Redmond et al. 2023). More recent duplications occurred in several fish and amphibian lineages.

Paralogous genes arising by genome duplication initially have functional redundancy; hence, long‐term retention is possible only if there is a selective advantage to having both copies. Reasons could include acquisition of new or supplementary roles, subdivision of pleiotropic roles, or specialization onto a subset of roles (Force et al. 1999; Marlétaz et al. 2018). In vertebrate evolution, retention of paralogues was commonest in genes encoding transcription factors and in genes with roles in development and signal transduction (Putnam et al. 2008). Reasons for these biases are unclear but may include modularity of gene regulatory elements giving potential for subfunctionalisation, multidomain structure of the encoded proteins, or greater scope for recruitment to novel roles. Hox gene clusters provide a case study, with most genes retained in multiple copies after genome duplications in vertebrate ancestry. For example, the human genome has 39 Hox genes spread across 4 Hox gene clusters descendent from a single cluster of 14 genes (Kuraku et al. 2008); this implies retention of 70% and loss of 30% of duplicated genes. Despite the high degree of retention, no mammalian Hox gene cluster has every paralogy group. Instead, there was a patchwork of retention leaving four partial clusters.

Using chromosome number as an indicator of polyploidy, it was suggested that whole genome duplication occurred at least three times in molluscan evolution (Hallinan and Lindberg 2011): within the Caenogastropoda (including cone snails and some other marine gastropods), the Stylommatophora (land snails and slugs) and the Cephalopoda (squid, octopus and relatives). Molecular and genomic analyses support the first two events, but not the third. Pardos‐Blas et al. (2021) provided the first strong support for a genome duplication in the order Neogastropoda, within the subclass Caenogastropoda, through sequencing and analysis of the genome of the Mediterranean cone snail, *Lautoconus ventricosus*. This revealed clear signatures of ancient genome duplication, including large blocks of intragenomic synteny. Farhat et al. (2023) provided additional support from the genome sequence of a second neogastropod *Stramonita haemastoma* and a member of the closely related Tonnoidea superfamily *Monoplex corrugatus*.

Within the Stylommatophora, extensive intragenomic synteny has been reported for snails in the genus *Lissachatina* and a slug in the genus *Arion*, consistent with an ancient genome duplication in land snails and slugs (Liu et al. 2021; Chen et al. 2022). The genome duplication has been suggested as partly facilitating adaptation to life on land within the gastropods. We were interested to know how genome duplication influenced the evolution of Hox gene clusters in Stylommatophora, particularly as the evolution of these genes has been well studied following genome duplication in vertebrates. Comparing the independent genome duplication events of Stylommatophora and vertebrates may reveal commonalities in the pathway of molecular evolution. Previous work has shown that molluscs without duplicated genomes generally have 11 Hox genes, named *Hox1*, *Hox2*, *Hox3*, *Hox4*, *Hox5*, *Lox5*, *Antp* (= *Hox7*), *Lox4*, *Lox2*, *Post2* and *Post1*, although individual genes have been lost in some species (Biscotti et al. 2014; Huan et al. 2020; Wollesen and Wanninger 2023). Within Stylommatophora, duplicated Hox gene clusters have been reported in *Lissachatina immaculata* and *L. fulica* (Liu et al. 2021). Here we use bioinformatic analyses and PCR to analyse Hox gene organization in 10 species of Stylommatophora and compare to other molluscs. We find duplicated but broken Hox gene clusters in all Stylommatophora and a patchwork retention of duplicate genes.

## 2 Results

### 2.1 Timing of Genome Duplication

**Figure 1:**
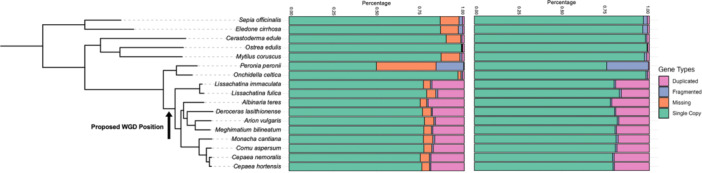
BUSCO analysis of mollusc genes. The central bar graph shows the percentage of single copy, duplicated, fragmented and missing copies of genes from a molluscan BUSCO set, related to a phylogenetic tree built from single copy genes. All phylogenetic nodes had 100% bootstrap support. The right‐hand bar graph shows the same data with missing genes excluded, used for PhylANOVA analyses of gene duplication.

*Lissachatina immaculata, L. fulica* and *Arion vulgaris* belong to deeply branching Stylommatophora clades Achatinina and Helicina. To examine if other members of the Stylommatophora likely share the same genome duplication, we ran BUSCO analyses on the genomes of 17 mollusc species using the mollusca_odb10 data set and examined copy number for the set of 5295 molluscan BUSCO genes across the species phylogeny. For non‐Stylommatophora, the proportion of duplicated genes in the data set was 0.54% to 1.5%. For the 10 species of Stylommatophora, the proportion ranged from 16% to 21% (Figure 1), significantly higher than in other molluscan clades tested (PhylANOVA *p* = 0.001; Supporting Information S1: Supplementary Data). These data are consistent with genome duplication on the lineage leading to Stylommatophora.

These analyses also permitted us to assess genome quality. We identified 10 species of Stylommatophora and one Systellommatophora with high quality genome assemblies (chromosomal‐level and 93.5% to 97.4% BUSCO completeness), and excluded one Systellommatophora scaffold‐level assembly from further analyses (*Peronia peronii*; 50.2% BUSCO completeness).

### 2.2 *Onchidella celtica*, Celtic Sea Slug

**Figure 2:**
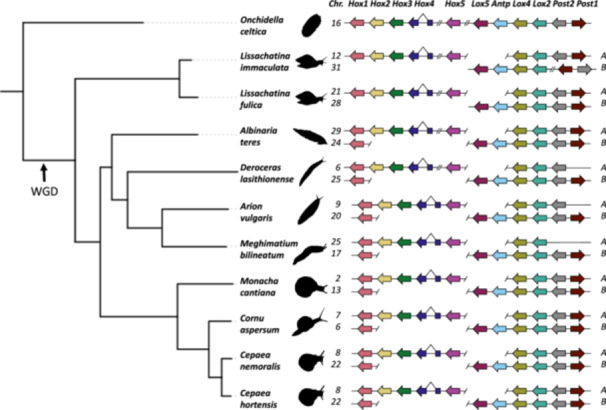
Genomic organization of Hox genes in 10 Stylommatophora and one outgroup. Arrows indicate transcriptional orientation, double‐hashed lines indicate large intergenic distances (> 1 Mb), and only introns within the homeobox are shown. To facilitate comparisons, homologous genes are lined up and distances are not to scale; entire subclusters may be inverted in some genomes (detailed in Supporting Information S1: Supplementary Data); Chr denotes chromosome number.

*Onchidella celtica* is a marine gastropod in the Systellommatophora and an outgroup to Stylommatophora; a chromosome‐level genome assembly is available (Darwin Tree of Life Project Consortium 2022). Using blast searches we found 11 Hox genes and classified these using diagnostic residues and phylogenetics into the 11 paralogy groups present in ancestral molluscs (Supplementary Data). All 11 Hox genes are on a single chromosome with the cluster broken into three sections: one has *Hox1*, *Hox2*, *Hox3* and *Hox4*, one has only *Hox5*, one has *Lox5, Antp*, *Lox4*, *Lox2*, *Post2* and *Post1* (Figure 2). We also deduce that there is an intron within the homeobox of *Hox4*.

### 2.3 *Lissachatina immaculata*, Giant African Land Snail

A genome sequence of *L. immaculata* was published by Liu et al. (2021); this report included demonstration of ancient genome duplication and two sets of Hox genes on two paralogous (self‐syntenic) chromosomes: 12 and 31. Our analysis agrees in overview but differs in detail. We find neither Hox gene clusters contains the full complement of 11 Hox genes. The more complete cluster, which we name HoxA, has 9 Hox genes and lacks paralogues of *Antp* and *Lox5* contrary to Liu et al. (2021). The less complete cluster, HoxB, has six genes, consistent with Liu et al. (2021). To test whether *Antp* and *Lox5* are indeed missing from HoxA we used degenerate PCR. We detect only single copies of *Antp* and *Lox5* – the HoxB paralogues – supporting our bioinformatic analysis. Splits in the clusters are in comparable positions to *O. celtica*. HoxA is split into three sections dispersed along the chromosome: one with *Hox1A*, *Hox2A*, *Hox3A* and *Hox4A* (with a homeobox intron), one contains only *Hox5A*, and one has *Lox4A*, *Lox2A*, *Post2A* and *Post1A*. In HoxB, one genomic region contains *AntpB*, *Lox5B*, *Lox4B* and *Lox2B*, with *Post2B* and *Post1B* separated (Figure 2). Hence, all 11 mollusc Hox paralogy groups are present but only four retained in duplicate.

### 2.4 *Lissachatina fulica*, Giant African Land Snail

A genome sequence of *L. fulica* is published (Guo et al. 2019). Our analysis of the reported genome sequence revealed putative frameshifts in the homeobox of *Hox1A*, *Hox3A* and *Post1B*. The *Antp* gene is not present in the genome assembly. To test validity of these findings, we extracted DNA from *L. fulica* and used PCR to amplify these four loci individually. Our sequenced PCR fragments contain intact reading frames, hence frameshifts in the assembly are likely errors. The missing *Antp* was successfully amplified and sequenced. Corrected PCR‐derived DNA sequences for *Hox1A*, *Hox3A*, *AntpB* and *Post1B* are given in Supplementary Data. The HoxA cluster is broken as in *L. immaculata* (Figure 2). Hence, all 11 mollusc Hox paralogy groups are present with four retained in duplicate.

### 2.5 *Albinaria teres*, Lasithian White‐Door Snail

A genome sequence for *Al. teres*, superfamily Clausilioidea, was assembled by the European Reference Genome Atlas via the Biodiversity Genomics Europe project: ERGA‐BGE (https://www.erga-biodiversity.eu, https://biodiversitygenomics.eu; McCartney et al. 2024). We find two Hox gene clusters, orthologous to HoxA and HoxB above. The organization is similar to *Lissachatina*, with splitting of clusters and dispersal along two chromosomes (Figure 2); the key difference is presence of *Hox1* in both HoxA and HoxB clusters. All 11 mollusc Hox paralogy groups are present with five retained in duplicate.

### 2.6 Three Species of Land Slug

Genome sequences were available for three species of land slug: *Deroceras lasithionense* (ERGA‐BGE; https://biodiversitygenomics.eu), *Arion vulgaris* (Chen et al. 2022) and *Meghimatium bileneatum* (Sun et al. 2024). These belong to a clade including Arionoidea and Limacoidea. We find near identical Hox gene organization in these species (*Me. bilineatum* apparently lacked *Post2A*; this could not be tested experimentally). In each case, there are two split Hox gene clusters, orthologous to HoxA and HoxB (Figure 2). All species have the two paralogues of *Hox1* as in *Al. teres* above. A key difference is consistent absence of the *Post1A* gene; consistency across three species suggests early loss of this locus in the evolution of land slugs. Hence, all 11 mollusc Hox paralogy groups are present with four retained in duplicate.

### 2.7 Four Species of Land Snail

**Figure 3:**
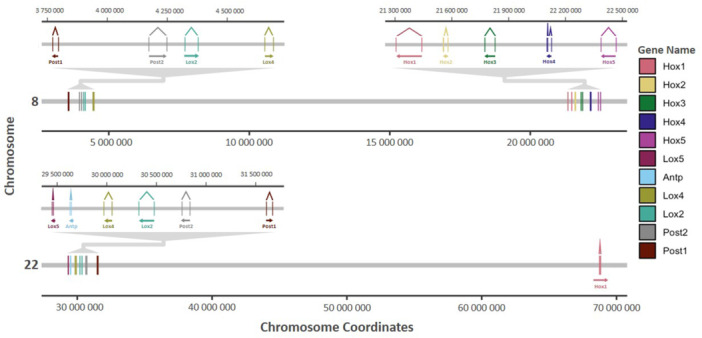
Hox gene clusters and subclusters of an example land snail species, *Cepaea nemoralis*, are shown to scale including predicted exons. Chromosomal locations in base pairs.

Genome sequences of four land snail species in the superfamily Helicoidea were made available by the Darwin Tree of Life project (Darwin Tree of Life Project Consortium 2022). These are *Monacha cantiana* (Kentish snail), *Cornu aspersum* (Garden snail), *Cepaea nemoralis* (Brown‐lipped snail), and *Cepaea hortensis* (White‐lipped snail). We also extracted genomic DNA from three species for PCR. Bioinformatic analysis of the genome sequences showed all four species have HoxA and HoxB, each dispersed along a chromosome. Duplicate *Hox1* genes were present, as in all land molluscs analyzed here apart from *Lissachatina*. Duplicate *Post1* genes were present, as in all land molluscs analyzed apart from slugs. Single *Antp* and *Lox5* were present, consistent with all other snails and slugs in our data set. The *Co*. *aspersum* reference assembly, but not the alternative haplotype, has a 12‐nucleotide deletion in the homeobox of *Hox1A*, disrupting its ability to fold into a homeodomain (Supporting Information S1: Supplementary Data). To test if this is a polymorphic variant, we used tblastn to search all available sequence read archive (SRA) datasets; we only recovered the intact homeobox, giving no support to the polymorphism hypothesis. We also extracted DNA from 6 specimens of *Co*. *aspersum*; PCR and sequencing revealed only the intact homeobox allele. The *Ce*. *hortensis* assembly analyzed lacks a *Post2* gene in the HoxA cluster; however, we successfully amplified and sequenced the *Post2A* homeobox in both *Cepaea* species: *Ce*. *nemoralis* and *Ce*. *hortensis* (Figure 2; Supporting Information S1: Supplementary Data). We show the Hox clusters of *Ce. nemoralis* to scale in Figure 3. Hence, all four species possess all 11 mollusc Hox paralogy groups with five retained in duplicate.

## 3 Discussion

Our bioinformatic and experimental analyses of Hox genes, and interrogation of BUSCO genes support the emerging picture that terrestrial slugs and snails experienced a complete genome duplication in their evolutionary history. These data indicate this occurred after the divergence of the fully terrestrial Stylommatophora from the air‐breathing marine and terrestrial Systellommatophora clade. The Stylommatophora analyzed here include representatives of both Achatinina and Helicina, two deeply branching lineages within the clade. We do not have data from the lineage leading to Scolodontina, the deepest branching Stylommatophora clade (Saadi and Wade 2019).

Our analyses of Hox genes in Stylommatophora give insights into how Hox genes evolved after genome duplication in terrestrial snails and slugs. First, all species descendent from the genome duplication retain two sets of Hox genes, located on two different chromosomes. We name these HoxA and HoxB. Second, HoxA and HoxB are both ‘broken clusters’. HoxA has a consistent split between *Hox5A* and *Lox4A*, and often additional breaks; in HoxB the *Hox1B* gene is separated. At least one break in the Hox gene cluster occurred before the genome duplication, between *Hox5* and *Lox5*, since we find this is shared with *Onchidella celtica* in the Systellommatophora. It is unclear whether this ancient breakage of the Hox cluster is associated with distinct regulation of the two ‘subclusters’ as proposed for the bivalve mollusc *Patinopecten yessoensis* (Wang et al. 2017).

Third, the location of *Hox5A* within the HoxA cluster appears surprisingly labile in the evolution of slugs and snails. In two slugs (genera *Arion* and *Meghimatium*) and four land snails (genera *Cornu*, *Cepaea*, *Monacha*), *Hox5A* remains in close proximity to *Hox4A* such that HoxA is only split into two subclusters. In contrast, in *Deroceras*, *Albinaria*, *Lissachatina* and the outgroup *Onchidella*, *Hox5A* has “escaped” this subcluster to differing degrees. This is least extreme in *Albinaria* where the intergenic distance is just over 1 Mb, and most dramatic in *Lissachatina* and *Onchidella* where the gene is chromosomally on the far side of *Post1A* (Supplementary Data). A possible explanation is that any selective constraint for maintaining a *Hox1A* to *Hox5A* subcluster, such as potential coregulation (Duboule 2007), is weakest for the *Hox5A* gene thereby permitting its repeated “escape.” A comparable example of escape from a Hox gene cluster is shown by the *Hoxb13* gene of vertebrates, a gene distantly separated from the otherwise tightly compacted Hoxb cluster. Functional analysis in mice and genetic association studies in sheep indicate that *Hoxb13* acquired a new role in controlling the extent of tail extension rather than positional specification (Economides et al. 2003; Li et al. 2023). Similar arguments for relaxation of selective constraint permitting gene dispersal have been proposed to explain why tight Hox gene clustering has been lost in invertebrates that do not determine rostrocaudal identities in a temporal sequence during development (Ferrier and Holland 2002; Duboule 2007).

Fourth, all species retain all 11 molluscan Hox paralogy groups, but neither HoxA nor HoxB has a complete set of 11 genes in any species. Nevertheless, the duplicated copies of *Lox4, Lox2,* and *Post2* are always retained, alongside two *Post1* and two *Hox1* in the majority of analyzed species. Interestingly, aside from *Hox1*, all commonly‐duplicated genes belong to the ‘middle‐to‐posterior’ region of the Hox cluster, spanning *Lox4* to *Post1* (Figure 2). The retention of an entire duplicated subcluster could hint at co‐option of a coregulated unit to a novel role.

Fifth, we infer the pathway of Hox gene loss. Genome duplication in Stylommatophora generated 22 Hox genes. This was followed by early loss of 6 genes: *Hox2B*, *Hox3B*, *Hox4B*, *Hox5B*, *Lox5A* and *AntpA*. Later in evolution, sporadic gene losses occurred, notably the deletion of *Hox1B* in *Lissachatina* or *Post1A* in some land slugs. This latter loss may be related to the reduction in the shells of these land slugs as *Post1A* has been extensively implicated in shell development (Samadi and Steiner 2009; Samadi and Steiner 2010; Huan et al. 2020); for example, shell reduction may have led to relaxed selection on this gene, permitting loss of function mutations and ultimately gene loss. Despite these losses, Hox genes had a high rate of retention after genome duplication in Stylommatophora. Terrestrial species analyzed have 15 or 16 Hox genes, implying a retention rate around 70%, comparable to that seen in vertebrates (Kuraku et al. 2008). We conclude that the patchwork pattern of Hox gene evolution was remarkably similar following independent genome duplications in vertebrates and terrestrial molluscs.

## 4 Methods

Genome sequences were downloaded from NCBI, except *L. fulica* from gigadb.org. Accession numbers for genomes and SRA data sets used are given in Supporting Information S1: Supplementary Data. All genomes were screened for 5295 BUSCO v.4 genes (Manni et al. 2021; mollusca_odb10.2019‐11‐20 data set https://busco.ezlab.org/list_of_lineages.html) and each classified as single copy, duplicated, fragmented or missing. Single‐copy BUSCOs common to all species were extracted using busco2fasta (github.com/lstevens17/busco2phylo‐nf), aligned using Mafft (Katoh 2005) and trimmed with ClipKIT (Steenwyk et al. 2020), then concatenated into a single matrix with PhyKIT (Steenwyk et al. 2020). A phylANOVA test from phytools R package was used to assess significance of differences in the proportion of duplicated BUSCOs between mollusc groups, after discounting missing genes, while accounting for evolutionary history (Revell 2012). To identify Hox genes, we used unannotated genome assemblies to overcome the possibility of automated annotation methods missing or mis‐annotating genes or pseudogenes. Hox gene sequences were found by iterative tblastn, searching with molluscan Hox sequences (Huan et al. 2020) and manual examination of all significant hits. Phylogenetic analysis using IQTree (Minh et al. 2020) and diagnostic residues described by Huan et al. (2020) were used to infer gene orthology. Exon 1 sequences for *Ce. nemoralis* were inferred using RNA‐seq data and then locations found by BLAST to genomic data. *Ce. hortensis* was collected from near Silchester, UK; specimens of *Lissachatina* were captive bred; specimens of *Ce. nemoralis* and *Co. aspersum* were collected from Oxford, UK. DNA was extracted using Qiagen DNeasy Blood and Tissue kit. PCR amplification was performed using Promega GoTaq DNA polymerase; reactions were directly sequenced using M13 and RM13 primers by Source BioScience, Cambridge, UK. Primer sequences, amplicon sequences, Hox coordinates, alignments, and phylogenetic analyses are given in Supplementary Data; sequences deposited on NCBI, accession numbers PV662042 to PV662053.

## Conflicts of Interest

The authors declare no conflicts of interest.

## Supporting information

Supporting Material Revision1.

## Data Availability

Genome and transcriptome sequence data that support part of this study are openly available in the National Center for Biotechnology Information (NCBI) database at https://www.ncbi.nlm.nih.gov/ or in GigaDB datasets at https://gigadb.org/; reference numbers are in Supplementary Data. Additional sequence data generated in this study are openly available in NCBI, accession numbers PV662042 to PV662053, and given in Supplementary Data.
