# Supplementary material for: Evolution of Duplicated Hox Gene Clusters in Land Snails and Slugs
Source: J Exp Zool B Mol Dev Evol. 2025 Aug 6;344(6):363–8. doi: 10.1002/jez.b.23322 (PMC12328838; doi:10.1002/jez.b.23322)

Evolution of duplicated Hox gene clusters in land snails and slugs

Finn McHale, Peter O Mulhair & Peter W H Holland

Supplementary Data

Contents

1. BUSCO analysis of gene duplication
2. Genomic locations of Hox genes
3. Genomes used for Hox gene analyses
4. Homeodomain alignments and orthology assignments
5. Phylogenetic analysis of homeodomains
6. PCR primer sequences
7. DNA sequences of amplified PCR fragments
8. Protein model of *Cornu aspernum* Hox1A alleles with and without deletion

1. **BUSCO analysis of duplicated genes in molluscs**. Predicted proteomes for each species were searched for a precomputed set of 5,295 'single copy' molluscan genes. This dataset was generated from non-Stylommatophora. (a) Phylogenetic tree of species analysed. The tree was determined by filtering the BUSCO set to retain only genes present in single copy all species. To the right of the phylogenetic tree, panel (i) shows the proportion of the generally single copy molluscan BUSCO genes that are duplicated in each species (purple), compared to single copy (green), missing genes (orange) and fragmented genes (blue). The *Peronia peronii* genome is a scaffold-level assembly of lower contiguity and completeness than chromosomal assemblies. Panel (ii) removes missing genes to facilitate comparison between single copy and duplicated genes. (b) Phylogenetically-aware ANOVA test (PhylANOVA) testing for excess of gene duplication in four molluscan clades; only clades with multiple species sampled can be tested. Table (i) shows pairwise t-tests comparing proportions of duplicated genes between four clades: Bivalvia, Cephalopoda, Systellomatophora and Stylommatophora. Table (ii) lists pairwise corrected p-values of these comparisons, showing significant differences only in the comparisons to Stylommatophora (p=0.006). Table (iii) summarises a separate PhylANOVA test, instead comparing proportions of duplicated genes between Stylommatophora and all non-Stylommatophora molluscs collectively, again showing Stylommatophora have a significantly higher proportion of duplicated genes (p=0.001). (c) Reconstruction of ancestral duplicated BUSCO proportions with duplication modelled as a continuous variable. Branch colour shows the proportion of duplicated genes modelled continuously and node pie-charts show the likelihood of the trait modelled as a binary trait (>/<10% duplicated). All reconstruction functions are found in the phytools R package (Revell 2012). Analyses show duplication is accelerated on the Stylommatophora branch. The dramatic difference between Stylommatophora and other molluscs in (a) and (c) is consistent with genome duplication.

(a) BUSCO analysis output

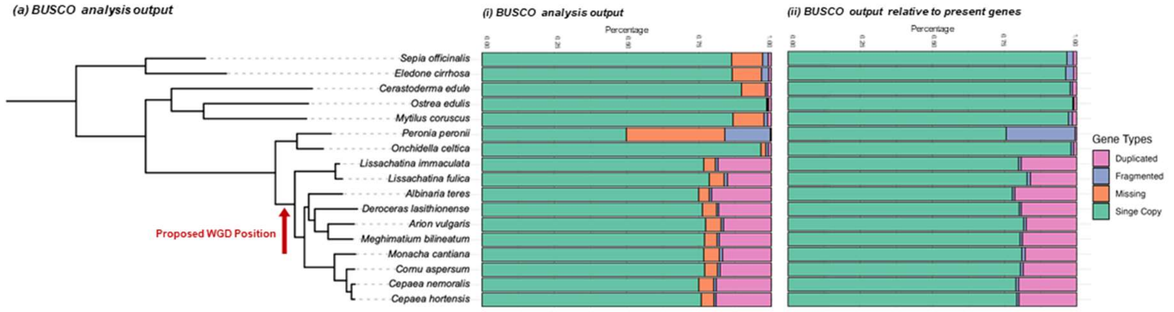

(b) PhylANOVA output comparing proportion of duplicated BUSCOs in Molluscan groups

(i) PhylANOVA pairwise t-values:

	Bivalvia	Cephalopoda	Systellommatophora	Stylommatophora
Bivalvia	0	0.164524	0.436855	**-20.88904**
Cephalopoda	-0.164524	0	0.248604	**-17.94616**
Systellommatophora	-0.436855	-0.248604	0	**-18.26711**
Stylommatophora	**20.889037**	**17.946161**	**18.267107**	0

(ii) PhylANOVA pairwise corrected p-values:

	Bivalvia	Cephalopoda	Systellommatophora	Stylommatophora
Bivalvia	1	1	1	**0.006**
Cephalopoda	1	1	1	**0.006**
Systellommatophora	1	1	1	**0.006**
Stylommatophora	**0.006**	**0.006**	**0.006**	1

(iii) PhylANOVA response table:

	Sum Sq	Mean Sq	F value	Pr(>F)
x	0.129856	0.129856	905.679068	**0.001**
Residual	0.002151	0.000143		

(c) Ancestral reconstruction of duplicated BUSCOs

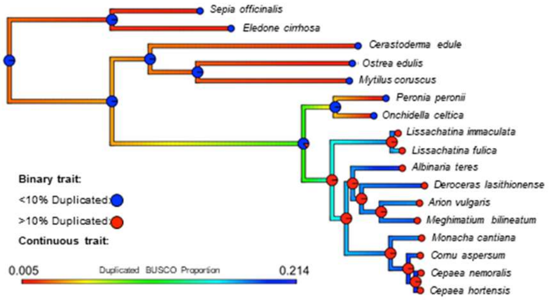

2. Genomic locations of Hox genes

Genomic locations of homeobox sequences for Hox genes in 10 Stylommatophora and one outgroup. In each case a single nucleotide position is given as a 'marker' to enable researchers to quickly locate each homeobox in the genome assemblies. When we found a gene by PCR (and verified by sequencing), but is not present in the genome assembly, this is indicated by 'PCR'.

Species / Gene	*Onchidella Celtica*			*Lissachatina immaculata*						*Lissachatina fulica*					
	Hox Cluster			Cluster A			Cluster B			Cluster A			Cluster B		
	Chromosome	Coordinate	Strand	Chromosome	Coordinate	Strand	Chromosome	Coordinate	Strand	Chromosome	Coordinate	Strand	Chromosome	Coordinate	Strand
Hox1	16	505063	+	12	40297792	-				21	38079633	+			
Hox2	16	375353	+	12	40509627	-				21	37862073	+			
Hox3	16	298686	+	12	40688315	-				21	37675437	+			
Hox4	16	165749	+	12	40951620	-				21	37400502	+			
Hox5	16	43236753	-	12	17508199	-				21	28678909	-			
Lox5	16	5939629	-				31	11907153	+				28	30694006	+
ANTP	16	6037833	-				31	11759973	+				*PCR*	*PCR*	*PCR*
Lox4	16	6232806	-	12	20089052	+	31	11496948	+	21	31310478	+	28	30287121	+
Lox2	16	6666183	-	12	19934092	+	31	11166422	+	21	31137221	+	28	29965131	+
Post2	16	6882374	-	12	19734507	+	31	4906970	-	21	30927861	+	28	29684574	+
Post1	16	7361476	+	12	19224641	-	31	5590113	+	21	30418583	-	28	29016985	-

Species / Gene	*Albinaria teres*						*Deroceras lasithionense*					
	Cluster A			Cluster B			Cluster A			Cluster B		
	Chromosome	Coordinate	Strand	Chromosome	Coordinate	Strand	Chromosome	Coordinate	Strand	Chromosome	Coordinate	Strand
Hox1	29	18096204	+	24	38637957	+	6	35012344	-	25	5346491	-
Hox2	29	17897158	+				6	35183387	-			
Hox3	29	17721825	+				6	35314283	-			
Hox4	29	17350407	+				6	35501988	-			
Hox5	29	16349513	+				6	33483323	+			
Lox5				24	17430826	-				25	17623139	+
ANTP				24	17584109	-				25	17503814	+
Lox4	29	596408	-	24	17798142	-	6	39393302	+	25	17278499	+
Lox2	29	704290	-	24	18042783	-	6	39325225	+	25	17054819	+
Post2	29	816967	-	24	18288387	-	6	39222167	+	25	16813649	+
Post1	29	998893	+	24	18868684	+				25	16284061	-

Species / Gene	*Arion vulgaris*						*Meghimatium bilineatum*					
	Cluster A			Cluster B			Cluster A			Cluster B		
	Chromosome	Coordinate	Strand	Chromosome	Coordinate	Strand	Chromosome	Coordinate	Strand	Chromosome	Coordinate	Strand
Hox1	9	56472940	-	20	27310929	-	25	7082315	-	17	6516370	-
Hox2	9	56669118	-				25	7266626	-			
Hox3	9	56832562	-				25	7424129	-			
Hox4	9	57044188	-				25	7627053	-			
Hox5	9	57258815	-				25	8519356	-			
Lox5				20	16717072	-				17	16250759	-
ANTP				20	16817462	-				17	16357366	-
Lox4	9	62262838	-	20	17054610	-	25	23642300	-	17	16634632	-
Lox2	9	62356713	-	20	17374616	-	25	23821747	-	17	16938839	-
Post2	9	62459248	-	20	17682786	-				17	17257634	-
Post1				20	18371482	+				17	18023185	+

Species / Gene	*Monacha cantiana*						*Cornu aspersum*					
	Cluster A			Cluster B			Cluster A			Cluster B		
	Chromosome	Coordinate	Strand	Chromosome	Coordinate	Strand	Chromosome	Coordinate	Strand	Chromosome	Coordinate	Strand
Hox1	2	122241459	+	13	29631314	+	7	23071608	-	6	113759838	+
Hox2	2	122040611	+				7	23314847	-			
Hox3	2	121896229	+				7	23545816	-			
Hox4	2	121648588	+				7	23864385	-			
Hox5	2	121374062	+				7	24240047	-			
Lox5				*CAXVRQ01000002*	*1601959*	+				6	78422101	-
ANTP				13	36584423	+				6	78614370	-
Lox4	2	132048794	-	13	36321413	+	7	7401286	-	6	78915541	-
Lox2	2	132169452	-	13	36028200	+	7	7083972	-	6	79290878	-
Post2	2	132344065	-	13	35738752	+	7	6848367	-	6	79731684	-
Post1	2	132633395	+	13	35085344	-	7	6258837	+	6	80777888	+

Species / Gene	*Cepaea nemoralis*						*Cepaea hortensis*					
	Cluster A			Cluster B			Cluster A			Cluster B		
	Chromosome	Coordinate	Strand	Chromosome	Coordinate	Strand	Chromosome	Coordinate	Strand	Chromosome	Coordinate	Strand
Hox1	8	21333322	-	22	68819221	+	8	106415939	+	22	18559800	-
Hox2	8	21583702	-				8	106159646	+			
Hox3	8	21801622	-				8	105939402	+			
Hox4	8	22132737	-				8	105653206	+			
Hox5	8	22413542	-				8	105299983	+			
Lox5				22	29326340	-				22	55440769	+
ANTP				22	29506688	-				22	55277909	+
Lox4	8	4477382	+	22	29847229	-	8	123496127	-	22	54982638	+
Lox2	8	4163947	+	22	30199662	-	8	123717953	-	22	54621046	+
Post2	8	4033761	+	22	30633973	-	*PCR*	*PCR*	*PCR*	22	54195719	+
Post1	8	3558424	-	22	31545450	+	8	124278154	+	22	53298044	-

3. **Genomes and transcriptome used for Hox gene analyses**

Lissachatina immaculata: GCA_009760885.1

Lissachatina fulica: Achatina_rebuild.fasta - GigaDB

Albinaria teres: GCA_964271275.1

Arion vulgaris: GCA_020796225.1

Cornu aspersum: GCA_964187895.1; alternative haplotype GCA_964187865.1

Cepaea hortensis: GCA_963921405.1

Cepaea nemoralis: GCA_964166675.1

Cepaea nemoralis transcriptome: GFLU00000000.1

Deroceras lasithionense: GCA_964271515.1

Meghamatium bilineatum: GCA_034231615.1

Monacha cantiana: GCA_964261925.1

Onchidella celtica: GCA_963931925.1

SRA data sets used to search for C. aspersum Hox1A 12 bp deletion:

SRX27177650, SRX27177649, SRX27177648, SRX26984990, ERX12326320, ERX12326318, ERX12326318, ERX12326322, ERX12326317, ERX12326319, ERX12354785, ERX12354787, ERX12354794, ERX12354790, ERX12354793, ERX12354791, ERX12354788, ERX12354786, ERX12354789, ERX12354792, SRX5115266, SRX5115265, SRX5115264, SRX2577257, SRX2546515, SRX1058255, SRX1058254, SRX1058252, SRX1058251, SRX1015093
= None of the hits had deletion

SRX16370107, SRX16370106, SRX16370105, SRX16370104, SRX16370103, SRX16370102, SRX14294989, SRX14294929, SRX14294729, SRX14294721, SRX14294547, SRX14294379, SRX14294378, SRX13463593, SRX13463592, SRX13463591, SRX10567816, SRX10567815, SRX5115283, SRX5115282, SRX5115281, SRX5115280, SRX5115279, SRX5115278, SRX5115277, SRX5115276, SRX5115275, SRX5115274, SRX5115273, SRX5115272, SRX5115271, SRX5115270, SRX5115269, SRX5115268, SRX5115267
= No hits

4. **Homeodomain alignments**. Alignments of deduced homeodomain sequences from Hox genes of Stylommatophora (this study) aligned to orthologous homeodomains from other molluscs (from Huan et al. 2020). Yellow highlighted amino acids are diagnostic residues for each paralogy group from Huan et al. (2020). Question marks indicate missing data; dashes indicate deletion (in allele of *C. aspersum* Hox1A in reference genome assembly). Red and blue amino acids in *Hox4* mark position of an intron, present in all 10 species of Stylommatophora analysed and in *Onchidella celtica* (Systellommatophora).

```
Lottia_Hox1              PNSGRTNFTNKQLTELEKEFHFNKYLTRARRIEIAASLGLNETQVKIWFQNRRMKQKKRM
Acanthochitona_Hox1      PNMGRTNFTNKQLTELEKEFHYNKYLTRARRIEIAASLGLNETQVKIWFQNRRMKQKKRM
Crassostrea_Hox1         PNMGRTNFTNKQLTELEKEFHFNKYLTRARRIEIAAALGLNETQVKIWFQNRRMKQKKRL
Antalis_Hox1             ANGGRTNFTNKQLTELEKEFHFNKYLTRARRIEIAAALGLNETQVKIWFQNRRMKQKKRM
Capitella_lab            PNMGRTNFTNKQLTELEKEFHFNKYLTRARRIEIAASLGLNETQVKIWFQNRRMKQKKRL
Terebratalia_lab         PNMGRTNFSNKQLTELEKEFHFNKYLTRARRIEIAAALGLNETQVKIWFQNRRMKQKKRM
Maculaura_lab            PNTGRTNFTNKQLTELEKEFHFNKYLTRARRIEIAAALGLNETQVKIWFQNRRMKQKKRM
L_immaculata_Hox1a       AGSGRTNFTNKQLTELEKEFHFNKYLTRARRIEIAAALGLNETQVKIWFQNRRMKQKKRM
L_fulica_Hox1a           ???GRTNFTNKQLTELEKEFHFNKYLTRARRIEIAAALGLNETQVKIWFQNRRMKQKKRM
A_teres_Hox1a            AGSGRTNFTNKQLTELEKEFHFNKYLTRARRIEIAAALGLNETQVKIWFQNRRMKQKKRM
A_teres_Hox1b            VASGRTNFTNKQLTELEKEFHFNKYLTRARRIEIAATLGLSETQIKIWFQNRRMKHKKRM
C_hortensis_Hox1a        AGSGRTNFTNKQLTELEKEFHFNKYLTRARRIEIAAALGLNETQVKIWFQNRRMKQKKRM
C_hortensis_Hox1b        AGSGRTNFTNKQLTELEKEFHFNKYLTRARRIEIAAALGLNETQVKIWFQNRRMKQKKRM
C_nemoralis_Hox1a        AGSGRTNFTNKQLTELEKEFHFNKYLTRARRIEIAAALGLNETQVKIWFQNRRMKQKKRM
C_nemoralis_Hox1b        AGSGRTNFTNKQLTELEKEFHFNKYLTRARRIEIAAALGLNETQVKIWFQNRRMKQKKRM
D_lasithionense_Hox1a    AGSGRTNFTNKQLTELEKEFHFNKYLTRARRIEIAAALGLNETQVKIWFQNRRMKQKKRM
D_lasithionense_Hox1b    MNSGRTHFTNKQVTELEKEFHFNKYLTRTRRLEISAALSLNETQVKIWFQNRRMKQKKRL
A_vulgaris_Hox1a         AGSGRTNFTNKQLTELEKEFHFNKYLTRARRIEIAAALGLNETQVKIWFQNRRMKQKKRM
A_vulgaris_Hox1b         TSAGRTNFTNKQLTELEKEFHFNKYLTRARRIEIAASLGLNETQVKIWFQNRRMKQKKRM
M_cantiana_Hox1a         AGSGRTNFTNKQLTELEKEFHFNKYLTRARRIEIAAALGLNETQVKIWFQNRRMKQKKRM
M_cantiana_Hox1b         AASGRTNFTNKQLTELEKEFHFNKYLTRARRIEIAAALGLNETQVKIWFQNRRMKQKKRM
C_aspersum_Hox1a-ref     AGSGRTNFTNKQLTELEKEFHFNKYLTRA----IAAALGLNETQVKIWFQNRRMKQKKRM
C_aspersum_Hox1a-alt     AGSGRTNFTNKQLTELEKEFHFNKYLTRARRIEIAAALGLNETQVKIWFQNRRMKQKKRM
C_aspersum_Hox1b         AGSGRTNFTNKQLTELEKEFHFNKYLTRARRIEIAAALGLNETQVKIWFQNRRMKQKKRM
M_bilineatum_Hox1a       AGSGRTNFTNKQLTELEKEFHFNKYLTRARRIEIAAALGLNETQVKIWFQNRRMKQKKRM
M_bilineatum_Hox1b       TAAGRTNFTNKQLTELEKEFHFNKYLTRARRIEIAASLGLNETQVKIWFQNRRMKQKKRM
O_celtica_Hox1           AGSGRTNFTNKQLTELEKEFHFNKYLTRARRIEIAAALGLNETQVKIWFQNRRMKQKKRM

Lottia_Hox2              SRRLRTAYTNTQLLELEKEFHFNKYLCRPRRIEIAASLDLTERQVKVWFQNRRMKYKRQS
Acanthochitona_Hox2      TRRLRTAYTNTQLLELEKEFHFNKYLCRPRRIEIAASLDLTERQVKVWFQNRRMKYKRQS
Crassostrea_Hox2         TRRLRTAYTNTQLLELEKEFHFNKYLCRPRRIEIAASLDLTERQVKVWFQNRRMKYKRQT
Antalis_Hox2             GRRLRTAYTNTQLLELEKEFHFNKYLCRPRRIEIAASLELTERQVKVWFQNRRMKYKRQT
Capitella_pb             PRRLRTAYTNTQLLELEKEFHENKYLCRPRRIEIAASLDLTERQVKVWFQNRRMKFKRQT
Terebratalia_pb          PRRLRTAYTNTQLLELEKEFHFNKYLCRPRRIEIAASLDLTERQVKVWFQNRRMKFKRQS
Maculaura_pb             PRRLRTAYTNSQLLELEKEFHFNKYLCRPRRIEIAASLDLTERQVKVWFQNRRMKYKRQS
L_immaculata_Hox2a       SRRLRTAYTNTQLLELEKEFHFNKYLCRPRRIEIAASLDLTERQVKVWFQNRRMKYKRQS
L_fulica_Hox2a           SRRLRTAYTNTQLLELEKEFHFNKYLCRPRRIEIAASLDLTERQVKVWFQNRRMKYKRQS
A_teres_Hox2a            SRRLRTAYTNTQLLELEKEFHFNKYLCRPRRIEIAASLDLTERQVKVWFQNRRMKYKRQS
C_hortensis_Hox2a        NRRLRTAYTNTQLLELEKEFHFNKYLCRPRRIEIAASLDLTERQVKVWFQNRRMKYKRQS
C_nemoralis_Hox2a        NRRLRTAYTNTQLLELEKEFHFNKYLCRPRRIEIAASLDLTERQVKVWFQNRRMKYKRQS
D_lasithionense_Hox2a    SRRLRTAYTNTQLLELEKEFHFNKYLCRPRRIEIAASLDLTERQVKVWFQNRRMKYKRQS
A_vulgaris_Hox2a         SRRLRTAYTNTQLLELEKEFHFNKYLCRPRRIEIAASLDLTERQVKVWFQNRRMKYKRQS
M_cantiana_Hox2a         NRRLRTAYTNTQLLELEKEFHFNKYLCRPRRIEIAASLDLTERQVKVWFQNRRMKYKRQS
C_aspersum_Hox2a         NRRLRTAYTNTQLLELEKEFHFNKYLCRPRRIEIAASLDLTERQVKVWFQNRRMKYKRQS
M_bilineatum_Hox2a       SRRLRTAYTNTQLLELEKEFHFNKYLCRPRRIEIAASLDLTERQVKVWFQNRRMKYKRQS
O_celtica_Hox2           SRRLRTAYTNTQLLELEKEFHFNKYLCRPRRIEIAASLELTERQVKVWFQNRRMKYKRQS

Lottia_Hox3              AKRARTAYTSAQLVELEKEFHFNRYLCRPRRIEMAALLNLSERQIKIWFQNRRMKFKKDC
Acanthochitona_Hox3      SKRARTAYTSAQLVELEKEFHFNRYLCRPRRIEMAALLSLTERQIKIWFQNRRMKFKKEQ
Crassostrea_Hox3         TKRARTAYTSAQLVELEKEFHFNRYLCRPRRIEMAALLSLTERQIKIWFQNRRMKFKKEQ
Antalis_Hox3             SKRARTAYTSAQLVELEKEFHFNRYLCRPRRIEMAALLNLTERQIKIWFQNRRMKFKKEQ
Capitella_Hox3           SKRARTAYTSAQLVELEKEFHFNRYLCRPRRIEMAALLNLTERQIKIWFQNRRMKYKKDQ
Terebratalia_Hox3        SKRARTAYTSAQLVELEKEFHFNRYLCRPRRIEMAALLSLSERQIKIWFQNRRMKFKKEQ
Maculaura_Hox3           PKRSRTAYTSAQLVELEKEFHFNRYLCRPRRIEMAALLNLSERQIKIWFQNRRMKYKKDQ
L_immaculata_Hox3a       SKRARTAYTSAQLVELEKEFHFNRYLCRPRRIEMAALLNLTERQIKIWFQNRRMKFKKEQ
L_fulica_Hox3a           SKRARTAYTSAQLVELEKEFHFNRYLCRPRRIEMAALLNLTERQIKIWFQNRRMKFKKEQ
A_teres_Hox3a            SKRARTAYTSAQLVELEKEFHFNRYLCRPRRIEMAALLNLTERQIKIWFQNRRMKFKKEQ
C_hortensis_Hox3a        SKRARTAYTSAQLVELEKEFHFNRYLCRPRRIEMAALLNLTERQIKIWFQNRRMKFKKEQ
C_nemoralis_Hox3a        SKRARTAYTSAQLVELEKEFHFNRYLCRPRRIEMAALLNLTERQIKIWFQNRRMKFKKEQ
D_lasithionense_Hox3a    SKRARTAYTSAQLVELEKEFHFNRYLCRPRRIEMAALLNLTERQIKIWFQNRRMKFKKEQ
A_vulgaris_Hox3a         SKRARTAYTSAQLVELEKEFHFNRYLCRPRRIEMAALLNLTERQIKIWFQNRRMKFKKEQ
M_cantiana_Hox3a         SKRARTAYTSAQLVELEKEFHFNRYLCRPRRIEMAALLNLTERQIKIWFQNRRMKFKKEQ
C_aspersum_Hox3a         SKRARTAYTSAQLVELEKEFHFNRYLCRPRRIEMAALLNLTERQIKIWFQNRRMKFKKEQ
M_bilineatum_Hox3a       SKRARTAYTSAQLVELEKEFHFNRYLCRPRRIEMAALLNLTERQIKIWFQNRRMKFKKEQ
O_celtica_Hox3           SKRARTAYTSAQLVELEKEFHFNRYLCRPRRIEMAALLNLTERQIKIWFQNRRMKFKKEQ
```

```
Lottia_Hox4                 SKRNRTAYTRHQVLELEKEFHFNRYLTRRRRIEIAHTLCLSERQIKIWFQNRRMKWKKEH
Acanthochitona_Hox4         TKRVRTAYTRHQVLELEKEFHFNRYLTRKRRIEIAHSLCLSERQIKIWFQNRRMKWKKEH
Crassostrea_Hox4            SKRNRTAYTRHQILELEKEFHFNRYLTRRRRIEIAHTLCLSERQIKIWFQNRRMKWKKEH
Antalis_Hox4                SKRNRTAYTRHQILELEKEFHFNRYLTRRRRIEIAHTLCLSERQIKIWFQNRRMKWKKEN
Capitella_Dfd               SKRTRTAYTRHQILELEKEFHFNRYLTRRRRIEIAHTLCLSERQIKIWFQNRRMKWKKEH
Terebratalia_Dfd            PKRSRTAYTRHQILELEKEFHFNRYLTRRRRIEIAHALCLTERQIKIWFQNRRMKWKKEH
Maculaura_Dfd               NKRTRTAYTRHQILELEKEFHFNRYLTRRRRIEIAHALCLTERQIKIWFQNRRMKWKKEH
L_immaculata_Hox4a          SKRNRTSYTRQQILEMEKEFHFNRYLTRRRRVEIANSLCLSERQIKIWFQNRRMKWKKEH
L_fulica_Hox4a              SKRNRTSYTRQQILEMEKEFHFNRYLTRRRRVEIANSLCLSERQIKIWFQNRRMKWKKEH
A_teres_Hox4a               SKRNRTSYSRQQILEMEKEFHYNRYLTRRRRIEIANSLNLSERQIKIWFQNRRMKWKKEH
C_hortensis_Hox4a           SKRNRTSYSRQQILEMEKEFHYNRYLTRRRRIEIASSLSLSERQVKIWFQNRRMKWKKEH
C_nemoralis_Hox4a           SKRNRTSYSRQQILEMEKEFHYNRYLTRRRRIEIASSLSLSERQVKIWFQNRRMKWKKEH
D_lasithionesne_Hox4a       SKRNRTSYSRQQILEMEKEFHYNRYLTRRRRIEIANSLSLSERQIKIWFQNRRMKWKKEH
A_vulgaris_Hox4a            SKRNRTSYSRQQILEMEKEFHYNRYLTRRRRIEIANSLSLSERQIKIWFQNRRMKWKKEH
M_cantiana_Hox4a            SKRNRTSYSRQQILEMEKEFHYNRYLTRRRRMEIASSLSLSERQVKIWFQNRRMKWKKEH
C_aspersum_Hox4a            SKRNRTSYSRQQILEMEKEFHYNRYLTRRRRIEIASSLSLSERQVKIWFQNRRMKWKKEH
M_bilineatum_Hox4a          SKRNRTSYSRQQILEMEKEFHYNRYLTRRRRIEIANSLTLSERQIKIWFQNRRMKWKKEH
O_celtica_Hox4              SKRNRTAYTRQQILELEKEFHFNRYLTRRRRIEIAHSLTLTERQIKIWFQNRRMKWKKEH

Lottia_Hox5                 SKRSRTSYTRHQTLELEKEFHYNKYLTRRRRIEIAHALNLTERQIKIWFQNRRMKWKKDH
Acanthochitona_Hox5         SKRSRTSYTRHQTLELEKEFHYNKYLTRRRRIEIAHSLNLTERQIKIWFQNRRMKWKKEH
Crassostrea_Hox5            SKRSRTSYTRHQTLELEKEFHYNKYLTRRRRIEIAHALNLTERQIKIWFQNRRMKWKKEH
Antalis_Hox5                TKRSRTSYTRHQTLELEKEFHYNKYLTRRRRIEIAHALNLTERQIKIWFQNRRMKWKKEH
Capitella_Scr               NKRTRTSYTRHQTLELEKEFHFNRYLTRRRRIEIAHSLNLTERQIKIWFQNRRMKWKKEH
Terebratalia_Scr            SKRTRTSYTRHQTLELEKEFHFNRYLTRRRRIEIAHALNLTERQIKIWFQNRRMKWKKEQ
Maculaura_Scr               SKRTRTSYTRYQTLELEKEFHFNRYLTRRRRIEIAHALNLTERQIKIWFQNRRMKWKKEQ
L_immaculata_Hox5a          TKRSRTSYTRHQTLELEKEFHYNKYLTRRRRIEIAHALNLTERQIKIWFQNRRMKWKKDH
L_fulica_Hox5a              TKRSRTSYTRHQTLELEKEFHYNKYLTRRRRIEIAHALNLTERQIKIWFQNRRMKWKKDH
A_teres_Hox5a               TKRSRTSYTRHQTLELEKEFHYNKYLTRRRRIEIAHALNLTERQIKIWFQNRRMKWKKDH
C_hortensis_Hox5a           TKRSRTSYTRHQTLELEKEFHYNKYLTRRRRIEIAHSLNLTERQIKIWFQNRRMKWKKDH
C_nemoralis_Hox5a           TKRSRTSYTRHQTLELEKEFHYNKYLTRRRRIEIAHSLNLTERQIKIWFQNRRMKWKKDH
D_lasithionense_Hox5a       TKRSRTSYTRHQTLELEKEFHYNKYLTRRRRIEIAHALNLTERQIKIWFQNRRMKWKKDH
A_vulgaris_Hox5a            SKRSRTSYTRHQTLELEKEFHYNKYLTRRRRIEIAHALNLTERQIKIWFQNRRMKWKKDH
M_cantiana_Hox5a            TKRSRTSYTRHQTLELEKEFHYNKYLTRRRRIEIAHSLNLTERQIKIWFQNRRMKWKKDH
C_aspersum_Hox5a            TKRSRTSYTRHQTLELEKEFHYNKYLTRRRRIEIAHSLNLTERQIKIWFQNRRMKWKKDH
M_bilineatum_Hox5a          NKRSRTSYTRHQTLELEKEFHYNKYLTRRRRIEIAHALNLTERQIKIWFQNRRMKWKKDH
O_celtica_Hox5              TKRSRTSYTRHQTLELEKEFHYNKYLTRRRRIEIAHALNLTERQIKIWFQNRRMKWKKDH

Lottia_Lox5                 QKRTRQTYTRYQTLELEKEFHFNRYLTRRRRIEVAHMLGLTERQIKIWFQNRRMKWKKEN
Acanthochitona_Lox5         TKRTRQTYTRHQTLELEKEFHYNRYLTRRRRIEIAHMLGLTERQIKIWFQNRRMKWKKEN
Crassostrea_Lox5            QKRTRQTYTRYQTLELEKEFHFNRYLTRRRRIEIAHLLGLTERQIKIWFQNRRMKWKKDN
Antalis_Lox5                QKRTRQTYTRYQTLELEKEFHFNRYLTRRRRIEIAHMLGLTERQIKIWFQNRRMKWKKEN
Capitella_Lox5              QKRTRQTYTRYQTLELEKEFHYNRYLTRRRRIEIAHALQLTERQIKIWFQNRRMKYKKEN
Terebratalia_Lox5           QKRTRQTYTRYQTLELEKEFHFNRYLTRRRRIEIAHALGLTERQIKIWFQNRRMKWKKEN
Maculaura_Lox5              QKRTRQTYTRYQTLELEKEFHFNKYLTRRRRIEIAHALGLTERQIKIWFQNRRMKWKKEN
L_immaculata_Lox5b          QKRTRQTYTRYQTLELEKEFHFNRYLTRRRRIEIAHMLGLTERQIKIWFQNRRMKWKKEN
L_fulica_Lox5b              ????RQTYTRYQTLELEKEFHFNRYLTRRRRIEIAHMLGLTERQIKIWFQNRRMKWKKEN
A_teres_Lox5b               QKRTRQTYTRYQTLELEKEFHFNRYLTRRRRIEIAHMLGLTERQIKIWFQNRRMKWKKEN
C_hortensis_Lox5b           QKRTRQTYTRYQTLELEKEFHFNRYLTRRRRIEIAHMLGLTERQIKIWFQNRRMKWKKEN
C_nemoralis_Lox5b           QKRTRQTYTRYQTLELEKEFHFNRYLTRRRRIEIAHMLGLTERQIKIWFQNRRMKWKKEN
D_lasithionense_Lox5b       QKRTRQTYTRYQTLELEKEFHFNRYLTRRRRIEIAHMLALTERQIKIWFQNRRMKWKKEN
A_vulgaris_Lox5b            QKRTRQTYTRYQTLELEKEFHFNRYLTRRRRIEIAHMLGLTERQIKIWFQNRRMKWKKEN
M_cantiana_Lox5b            QKRTRQTYTRYQTLELEKEFHFNRYLTRRRRIEIAHMLGLTERQIKIWFQNRRMKWKKEN
C_aspersum_Lox5b            QKRTRQTYTRYQTLELEKEFHFNRYLTRRRRIEIAHMLGLTERQIKIWFQNRRMKWKKEN
M_bilineatum_Lox5b          QKRTRQTYTRYQTLELEKEFHFNRYLTRRRRIEIAHMLGLTERQIKIWFQNRRMKWKKEN
O_celtica_Lox5              QKRTRQTYTRYQTLELEKEFHFNRYLTRRRRIEIAHMLGLTERQIKIWFQNRRMKWKKEN

Lottia_ANTP                 RKRGRQTYTRYQTLELEKEFHFNRYLTRRRRIEIAHALCLTERQIKIWFQNRRMKWKKEA
Acanthochitona_ANTP         RKRGRQTYTRYQTLELEKEFHFNKYLTRRRRIEIAHALCLTERQIKIWFQNRRMKWKKEN
Capitella_ANTP              RKRGRQTYTRYQTLELEKEFHFNRYLTRRRRIEIAHALCLTERQIKIWFQNRRMKWKKEN
Terebratalia_ANTP           RKRGRQTYSRHQTLELEKEFHFNRYLTRRRRIEIAHALCLTERQIKIWFQNRRMKWKKEN
Maculaura_ANTP              RKRGRQTYTRYQTLELEKEFHFNKYLTRRRRIETAHALCLTERQIKIWFQNRRMKWKKEN
L_immaculata_ANTPb          RKRGRQTYTRYQTLELEKEFHYNRYLTRRRRIEIAHSLCLTERQIKIWFQNRRMKWKKEC
L_fulica_ANTPb              ???GRQTYTRYQTLELEKEFHYNRYLTRRRRIEIAHSLCLTERQIKIWFQNRRMKWKKEC
A_teres_ANTPb               PSRGRQTYTRYQTLELEKEFHYNRYLTRRRRIEISHSLCLTERQIKIWFQNRRMKWKKEC
C_hortensis_ANTPb           RKRGRQTYTRYQTLELEKEFHYNRYLTRRRRIEIAHSLCLTERQIKIWFQNRRMKWKKEC
C_nemoralis_ANTPb           RKRGRQTYTRYQTLELEKEFHYNRYLTRRRRIEIAHSLCLTERQIKIWFQNRRMKWKKEC
D_lasithionense_ANTPb       RKRGRQTYTRFQTLELEKEFHYNRYLTRRRRIEIAHCLCLTERQIKIWFQNRRMKWKKEC
```

```
A_vulgaris_ANTPb          RKRGRQTYTRYQTLELEKEFHYNRYLTRRRRIEIAHSLCLTERQIKIWFQNRRMKWKKEC
M_cantiana_ANTPb          RKRGRQTYTRFQTLELEKEFHYNRYLTRRRRIEIAHSLCLTERQIKIWFQNRRMKWKKES
C_aspersum_ANTPb          RKRGRQTYTRYQTLELEKEFHYNRYLTRRRRIEIAHSLCLTERQIKIWFQNRRMKWKKEC
M_bilineatum_ANTPb        HKRGRQTYTRYQTLELEKEFHYNRYLTRRRRIEIAHTLCLSERQIKIWFQNRRMKWKKEC
O_celtica_antp            RKRGRQTYTRYQTLELEKEFHYNRYLTRRRRIEIAHSLCLTERQIKIWFQNRRMKWKKEC

Lottia_Lox4               RRRGRQTYSRYQTLELEKEFQFNHYLTRKRRIEIAHTLCLTERQIKIWFQNRRMKMKKER
Acanthochitona_Lox4       RRRGRQTYSRYQTLELEKEFQFNHYLTRKRRIEVAHALCLTERQIKIWFQNRRMKLKKER
Crassostrea_Lox4          RRRGRQTYSRYQTLELEKEFQFNHYLTRKRRIEVAHSLCLTERQIKIWFQNRRMKLKKER
Antalis_Lox4              RRRGRQTYSRYQTLELEKEFQFNHYLTRKRRIEIAHSLCLTERQIKIWFQNRRMKLKKEK
Capitella_Lox4            RRRGRQTYSRYQTLELEKEFQFNHYLTRKRRIEIAHALCLTERQIKIWFQNRRMKLKKER
Terebratalia_Lox4         RRRGRQTYSRYQTLELEKEFQFNHYLTRKRRIEVAHALCLTERQIKIWFQNRRMKLKKER
Maculaura_Lox4            RRRGRQTYSRYQTLELEKEFQFNHYLTRRRRIEIAHSLCLTERQIKIWFQNRRMKLKKER
L_immaculata_Lox4a        RRRGRQTYSRYQTLELEKEFQFNHYLTRKRRIEIAHTLCLTERQIKIWFQNRRMKLKKEK
L_immaculata_Lox4b        RRRGRQTYSRYQTLELEKEFQFNHYLTRKRRIEIAHSLCLTERQIKIWFQNRRMKLKKEK
L_fulica_Lox4a            RRRGRQTYSRYQTLELEKEFQFNHYLTRKRRIEIAHTLCLTERQIKIWFQNRRMKLKKEK
L_fulica_Lox4b            RRRGRQTYSRYQTLELEKEFQFNHYLTRKRRIEIAHSLCLTERQIKIWFQNRRMKLKKEK
A_teres_Lox4a             RRRGRQTYSRFQTLELEKDFQFNNYLSRKRRVEISHSLCLTERQVKIWFQNRRMKLKKDK
A_teres_Lox4b             RRRGRQTYSRYQTLELEKEFQFNHYLTRKRRIEIAHSLCLTERQIKIWFQNRRMKLKKEK
C_hortensis_Lox4a         RRRGRQTYSRLQTLELEKEFQFNHYLTRRRRGEIADSLCLSERQIKIWFQNRRMKMKKEK
C_hortensis_Lox4b         RRRGRQTYSRYQTLELEKEFQFNHYLTRKRRIEIAHSLCLTERQIKIWFQNRRMKLKKEK
C_nemoralis_Lox4a         RRRGRQTYSRLQTLELEKEFQFNHYLTRRRRGEIADSLCLSERQIKIWFQNRRMKMKKEK
C_nemoralis_Lox4b         RRRGRQTYSRYQTLELEKEFQFNHYLTRKRRIEIAHSLCLTERQIKIWFQNRRMKLKKEK
D_lasithionense_Lox4a     RRRGRQTYSRHQTLELEKEFQFNNYLTRKRRMEIALSLSLTERQIKIWFQNRRMKLKKDK
D_lasithionense_Lox4b     RRRGRQTYSRYQTLELEKEFQFNHYLTRKRRIEIAHSLCLTERQIKIWFQNRRMKLKKEK
A_vulgaris_Lox4a          RRRGRQTYSRYQTLELEKDFQFSHYLTRKRRVEIAESLCLSERQVKIWFQNRRMKLKKEK
A_vulgaris_Lox4b          RRRGRQTYSRYQTLELEKEFQFNHYLTRKRRIEIAHSLCLTERQIKIWFQNRRMKLKKEK
M_cantiana_Lox4a          RRRGRQTYSRLQTLELEKEFQFSYYLTRRRRGEIADTLRLSERQIKIWFQNRRMKMKKEK
M_cantiana_Lox4b          RRRGRQTYSRYQTLELEKEFQFNHYLTRKRRIEIAHSLCLTERQIKIWFQNRRMKLKKEK
C_aspersum_Lox4a          RRRGRQTYSRYQTLELEKEFQFNHYLTRKRRIEIAHSLCLTERQIKIWFQNRRMKLKKEK
C_aspersum_Lox4b          RCRGRQTYSRLQTLELEKEFQFNHYLTRRRRAEIADSLCLSERQIKIWFQNRRMKMKKEK
M_bilineatum_Lox4a        RRRGRQTYSRYQTLELEKEFQFNHYLTRKRRMEISHLLCLSERQIKIWFQNRRMKLKKEK
M_bilineatum_Lox4b        RRRGRQTYSRYQTLELEKEFQFNHYLTRKRRIEIAHSLCLTERQIKIWFQNRRMKLKKEK
O_celtica_Lox4            RRRGRQTYSRYQTLELEKEFQFNHYLTRKRRIEIAHSLCLTERQIKIWFQNRRMKLKKEK

Lottia_Lox2               RRRGRQTYTRFQTLELEKEFKFNRYLTRRRRIELSHMLCLTERQIKIWFQNRRMKEKKEL
Acanthochitona_Lox2       RRRGRQTYTRFQTLELEKEFKFNRYLTRRRRIELSHMLCLTERQIKIWFQNRRMKEKKEL
Crassostrea_Lox2          RRRGRQTYTRFQTLELEKEFKFNRYLTRRRRIELSHMLCLTERQIKIWFQNRRMKEKKEL
Antalis_Lox2              RRRGRQTYTRYQTLELEKEFKFNRYLTRRRRIELSHMLCLTERQIKIWFQNRRMKEKKEL
Capitella_Lox2            RRRGRQTYTRYQTLELEKEFKFNRYLTRRRRIELSHMLCLTERQIKIWFQNRRMKEKKEI
L_immaculata_Lox2a        RRRGRQTYTRFQTLELEKEFKFNRYLTRRRRIELSHMLCLTERQIKIWFQNRRMKEKKEL
L_immaculata_Lox2b        RRRGRQTYTRFQTLELEKEFKFNRYLTRRRRIELSHMLCLTERQIKIWFQNRRMKEKKEL
L_fulica_Lox2a            RRRGRQTYTRFQTLELEKEFKFNRYLTRRRRIELSHMLCLTERQIKIWFQNRRMKEKKEL
L_fulica_Lox2b            RRRGRQTYTRFQTLELEKEFKFNRYLTRRRRIELSHMLCLTERQIKIWFQNRRMKEKKEL
A_teres_Lox2a             RRRGRQTYTRFQTLELEKEFKFNRYLTRRRRIELSHMLCLTERQIKIWFQNRRMKEKKEL
A_teres_Lox2b             RRRGRQTYTRFQTLELEKEFKFNRYLTRRRRIELSHMLCLTERQIKIWFQNRRMKEKKEL
C_hortensis_Lox2a         RRRGRQTYTRFQTLELEKEFKFNRYLTRRRRIELSHMLCLTERQIKIWFQNRRMKEKKEL
C_hortensis_Lox2b         RRRGRQTYTRFQTLELEKEFKFNRYLTRRRRIELSHMLCLTERQIKIWFQNRRMKEKKEL
C_nemoralis_Lox2a         RRRGRQTYTRFQTLELEKEFKFNRYLTRRRRIELSHMLCLTERQIKIWFQNRRMKEKKEL
C_nemoralis_Lox2b         RRRGRQTYTRFQTLELEKEFKFNRYLTRRRRIELSHMLCLTERQIKIWFQNRRMKEKKEL
D_lasithionense_Lox2a     RRRGRQTYTRFQTLELEKEFKFNRYLTRRRRIELSHMLCLTERQIKIWFQNRRMKEKKEL
D_lasithionense_Lox2b     RRRGRQTYTRFQTLELEKEFKFNRYLTRRRRIELSHMLCLTERQIKIWFQNRRMKEKKEL
A_vulgaris_Lox2a          RRRGRQTYTRYQTLELEKEFKFNRYLTRRRRIELSHMLCLTERQIKIWFQNRRMKEKKEL
A_vulgaris_Lox2b          RRRGRQTYTRFQTLELEKEFKFNRYLTRRRRIELSHMLCLTERQIKIWFQNRRMKEKKEL
M_cantiana_Lox2a          RRRGRQTYTRFQTLELEKEFKFNRYLTRRRRIELSHMLCLTERQIKIWFQNRRMKEKKEL
M_cantiana_Lox2b          RRRGRQTYTRFQTLELEKEFKFNRYLTRRRRIELSHMLCLTERQIKIWFQNRRMKEKKEL
C_aspersum_Lox2a          RRRGRQTYTRFQTLELEKEFKFNRYLTRRRRIELSHMLCLTERQIKIWFQNRRMKEKKEL
C_aspersum_Lox2b          RRRGRQTYTRFQTLELEKEFKFNRYLTRRRRIELSHMLCLTERQIKIWFQNRRMKEKKEL
M_bilineatum_Lox2a        RRRGRQTYTRYQTLELEKEFKFNRYLTRRRRIELSHMLCLTERQIKIWFQNRRMKEKKEL
M_bilineatum_Lox2b        RRRGRQTYTRFQTLELEKEFKFNRYLTRRRRIELSHMLCLTERQIKIWFQNRRMKEKKEL
O_celtica_Lox2            RRRGRQTYTRFQTLELEKEFKFNRYLTRRRRIELSHMLCLTERQIKIWFQNRRMKEKKEL

Lottia_Post2              GRKKRKPYTRYQTMVLENEFLSSSYITRQKRWEISCKLQLSERQVKVWFQNRRMKRKKLT
Acanthochitona_Post2      GRKKRKPYTRYQTMVLENEFLNNSYITROKRWEISCKLQLTERQVKVWFQNRRMKRKKLN
Crassostrea_Post2         GRKKRKPYTRYQTMVLENEFLNSSYITRQKRWEISCKLQLSERQVKVWFQNRRMKRKKLN
Antalis_Post2             GRKKRKPYTRYQTMVLENEFLNSSYITRQKRWEISCKLQLSERQVKVWFQNRRMKRKKLN
Capitella_Post2           QRKKRKPYTRYQTMVLENEFINNSYITRQKRWEISCKLHLSERQVKVWFQNRRMKRKKLN
Terebratalia_Post2        SRKKRKPYTRYQNMLLENEFIANSYITRQKRWEISCKLQLTERQVKVWFQNRRMKRKKLT
```

```
Maculaura_Post2          TRKKRKPYTRYQTMVLENEFMTNSYITRQKRWEISCKLHLTERQVKVWFQNRRMKRKKLN
L_immaculata_Post2a      GRKKRKPYTRYQTMVLENEFLSSSYITRQKRWEISCKLQLSERQVKVWFQNRRMKRKKLT
L_immaculata_Post2b      GRKKRKPYTRYQTMVLENEFLNSSYITRQKRWEISCKLQLSERQVKVWFQNRRMKRKKLT
L_fulica_Post2a          GRKKRKPYTRYQTMVLENEFLSSSYITRQKRWEISCKLQLSERQVKVWFQNRRMKRKKLT
L_fulica_Post2b          GRKKRKPYTRYQTMVLENEFLNSSYITRQKRWEISCKLQLSERQVKVWFQNRRMKRKKLT
A_teres_Post2a           GRKKRKPYTRYQTMVLENEFLTSSYITRQKRWEISCKLQLSERQVKVWFQNRRMKRKKLT
A_teres_Post2b           GRKKRKPYTRYQTMVLENEFLNSSYITRQKRWEISCKLQLSERQVKVWFQNRRMKRKKLT
C_hortensis_Post2a       ????RKPYTRYQTMVLENEFLSSSYITRQKRWEISCKLQLSERQVKVWFQNRRMKRKKLT
C_hortensis_Post2b       GRKKRKPYTRYQTMVLENEFLNSSYITRQKRWEISCKLQLSERQVKVWFQNRRMKRKKLT
C_nemoralis_Post2a       GRKKRKPYTRYQTMVLENEFLNSSYITRQKRWEISCKLQLSERQVKVWFQNRRMKRKKLT
C_nemoralis_Post2b       GRKKRKPYTRYQTMVLENEFLNSSYITRQKRWEISCKLQLSERQVKVWFQNRRMKRKKLT
D_lasithionense_Post2a GRKKRKPYSRYQTMVLENEFLTSSYITRQKRWEISCKLQLSERQVKVWFQNRRMKRKKLT
D_lasithionense_Post2b GRKKRKPYTRYQTMVLENEFLNSSYITRQKRWEISCKLQLSERQVKVWFQNRRMKRKKLT
A_vulgaris_Post2a        GRKKRKPYTRYQTMVLENEFLTSSYITRQKRWEISCKLQLSERQVKVWFQNRRMKRKKLT
A_vulgaris_Post2b        GRKKRKPYTRYQTMVLENEFLNSSYITRQKRWEISCKLQLSERQVKVWFQNRRMKRKKLT
M_cantiana_Post2a        GRKKRKPYTRYQTMVLENEFLSSSYITRQKRWEISCKLQLSERQVKVWFQNRRMKRKKLT
M_cantiana_Post2b        GRKKRKPYTRYQTMVLENEFLNSSYITRQKRWEISCKLQLSERQVKVWFQNRRMKRKKLT
C_aspersum_Post2a        GRKKRKPYTRYQTMVLENEFLSSSYITRQKRWEISCKLQLSERQVKVWFQNRRMKRKKLT
C_aspersum_Post2b        GRKKRKPYTRYQTMVLENEFLNSSYITRQKRWEISCKLQLSERQVKVWFQNRRMKRKKLT
M_bilineatum_Post2b      GRKKRKPYTRYQTMVLENEFLNSSYITRQKRWEISCKLQLSERQVKVWFQNRRMKRKKLT
O_celtica_Post2          GRKKRKPYTRYQTMVLENEFLNSSYITRQKRWEISCKLQLSERQVKVWFQNRRMKRKKLT

Lottia_Post1             LRKRRRPYSKFQIAELEREYNGSTYVSKSRRWELSQLINLSERQIKIWFQNRRIKAKKII
Crassostrea_Post1        LRKRRRPYSKFQIAELEREYNNSTYISKSRRWELSQLINLSERQIKIWFQNRRIKAKKVS
Antalis_Post1            LRKRRRPYSKFQIAELEREYANSTYISKSRRWELSQLINLSERQIKIWFQNRRIKAKKIQ
Capitella_Post1          PKKKRKPYSKPQVSALENEYSTSTYITKARRKEVARELDLTERQIKIWYQNRRIKEKKIA
Terebratalia_Post1       MRKRRKPYSKQQINELEREYVKTTYISKPKRWELAQRLNLSERQVKIWFQNRRMKEKKMR
L_immaculata_Post1a      LRKRRRPYSKFQIAELEREYTSSTYISKSRRWELSQLINLSERQIKIWFQNRRIKAKKLQ
L_immaculata_Post1b      LRKRRRPYSKFQIAELEREYASSTYISKSRRWELSQLINLSERQIKIWFQNRRIKAKKLQ
L_fulica_Post1a          LRKRRRPYSKFQIAELEREYTSSTYISKSRRWELSQLINLSERQIKIWFQNRRIKAKKLQ
L_fulica_Post1b          LRKRRRPYSKFQIAELEREYASSTYISKSRRWELSQLINLSERQIKIWFQNRRIKAKKLQ
A_teres_Post1a           LRKRRRPYSKLQIAELEREYSSSTYISKSRRWELSQLINLSERQIKIWFQNRRIKAKKLQ
A_teres_Post1b           LRKRRRPYSKFQIAELEREYASSTYISKSRRWELSQLINLSERQIKIWFQNRRIKAKKLQ
C_hortensis_Post1a       LKKRRRPYSKLQIAELEREYSSSTYISKSRRWELSQIINLSERQIKIWFQNRRIKAKKIQ
C_hortensis_Post1b       LRKRRRPYSKFQIAELEREYASSTYISKSRRWELSQLINLSERQIKIWFQNRRIKAKKLQ
C_nemoralis_Post1a       LKKRRRPYSKLQIAELEREYSSSTYISKSRRWELSQIISLSERQIKIWFQNRRIKAKKIQ
C_nemoralis_Post1b       LRKRRRPYSKFQIAELEREYASSTYISKSRRWELSQLINLSERQIKIWFQNRRIKAKKLQ
D_lasithionense_Post1b LRKRRRPYSKFQIAELEREYASSTYISKSRRWELSQLINLSERQIKIWFQNRRIKAKKLQ
A_vulgaris_Post1b        LRKRRRPYSKFQIAELEREYASSTYISKSRRWELSQLINLSERQIKIWFQNRRIKAKKLQ
M_cantiana_Post1a        LKKRRRPYSKLQIAELEREYSGSTYISKSRRWELSQLISLSERQIKIWFQNRRIKAKKIH
M_cantiana_Post1b        LRKRRRPYSKFQIAELEREYASSTYISKSRRWELSQLINLSERQIKIWFQNRRIKAKKLQ
C_aspersum_Post1a        LKKRRRPYSKLQIAELEREYSSSTYISKSRRWELSQIINLSERQIKIWFQNRRIKAKKIQ
C_aspersum_Post1b        LRKRRRPYSKFQIAELEREYASSTYISKSRRWELSQLINLSERQIKIWFQNRRIKAKKLQ
M_bilineatum_Post1b      LRKRRRPYSKFQIAELEREYASSTYISKSRRWELSQLINLSERQIKIWFQNRRIKAKKLQ
O_celtica_Post1          LRKRRRPYSKFQIAELEREYASSTYISKSRRWELSQLINLSERQIKIWFQNRRIKAKKLQ
```

5. **Phylogenetic analysis of homeodomains**. Maximum likelihood tree of homeodomains only, built using the fast search algorithm of IQ-Tree (Nguyen HA et al. 2015. *Molecular Biology and Evolution* 32: 268–274). Midpoint rooted.

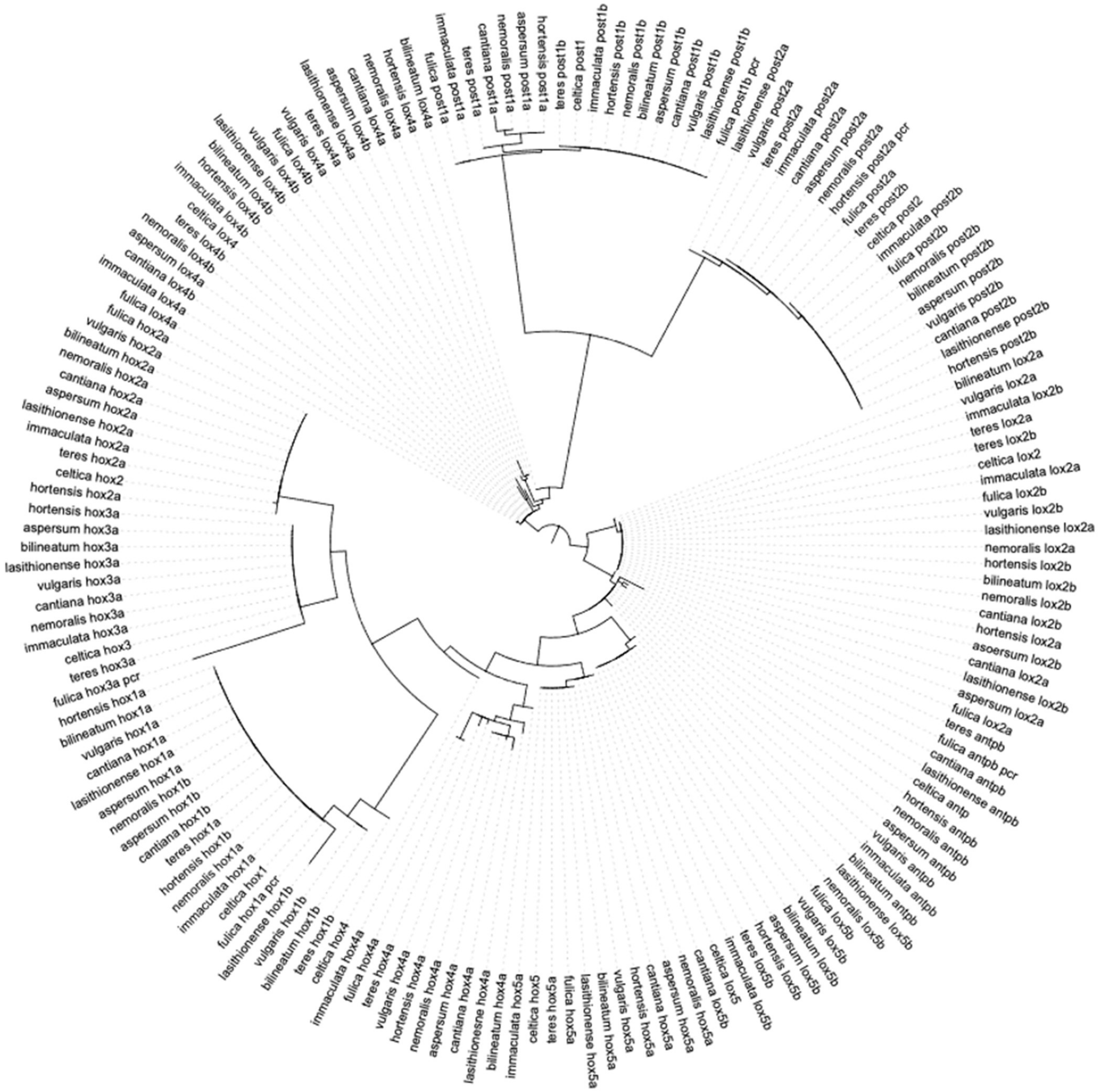

6. PCR primer sequences

Blue text indicates nucleotides added to provide M13 and RM13 primer sites for sequencing.

Lissachatina ANTP degenerate primers designed to amplify all Antp:
F: TGTAAAACGACGGCCAGTGGCCGGCARACNTAYAC
R: CAGGAAACAGCTATGACCTGCAGCTGYTTRCAYTC

Lissachatina Lox5 degenerate primers designed to amplify all Lox5:
F: TGTAAAACGACGGCCAGTAGGACACGGCARACNTAYAC
R: CAGGAAACAGCTATGACTCACAGCTTTTGTAGRTTRTTYTC

L. fulica Hox1A:
F: TGTAAAACGACGGCCAGTAAATCAACAGAATTCTCCAACAC
R: CAGGAAACAGCTATGACACACAACGCAAGATTCGGAAC

L. fulica Hox3A:
F: TGTAAAACGACGGCCAGTTCAAAGCGCGCTCGCACAGC
R: CAGGAAACAGCTATGACAAGCTTTGGTATGTAATCATATGAGCCCTG

L. fulica Post1B:
F: TGTAAAACGACGGCCAGTCGCCCATATTCAAAATTCCAAATCGC
R: CAGGAAACAGCTATGACGGAAACGGCGATCGCAGAAGG

L. fulica ANTPB:
F: TGTAAAACGACGGCCAGTAAAAGAGGCCGGCAGACCTAYAC
R: CAGGAAACAGCTATGACTTTCTCGGGCGTACATTCGCCRTTDAT

Cornu aspersum Hox1a:
F: TGTAAAACGACGGCCAGTGCCGGTTCGGGTCGCACAAACTT
R: CAGGAAACAGCTATGACCCTAGTGTCATGACATAGCGCCAGATT

Cepaea hortensis Post2A:
F: TGTAAAACGACGGCCAGTCGGAAACCTTACACCAGGTAYCA
R: CAGGAAACAGCTATGACTATGATTGGTCCATTCTTRTGYTC

7. DNA sequences of amplified PCR fragments

NCBI GenBank accession numbers for the sequences below: PV662043 to PV662053

L. fulica ANTP-B:
Primers: L. fulica ANTPB
ELEKEFHYNRYLTRRRRIEIAHSLCLTERQIKIWFQNRRMKWKKECKQLQI
gaactagaaaaggaattccattacaatcgatacctaacccgccgacggcgtatcgaaatcgcccactccctgtgt
ctgacggagaggcagataaagatatggttccagaacagaagaatgaagtggaagaaggagtgtaaacagttgcag
atc

L. fulica ANTP:
Primers: Lissachatina ANTP degenerate
RYQTLELEKEFHYNRYLTRRRRIEIAHSLCLTERQIKIW
ccatatctttatctgcctctccgtcagacacagggagtgggcgatttcgatacgccgtcggcgggttaggtatcg
attgtaatggaattccttttctagttccagggtttggtatct

L. fulica Lox5:
Primers: Lissachatina Lox5 degenerate
ELEKEFHFNRYLTRRRRIEIAHMLGLTERQIKIWFQNRRMKWKK
gagctggagaaggaattccactttaaccgctacctaactcgccgccgtcgtatagagattgcccacatgctgggg

ctgacggaaagacagattaagatttggttccagaaccggagaatgaaatggaaaaagg

L. fulica Hox1a:
Primers: L. fulica Hox1a
RTNFTNKQLTELEKEFHFNKYLTRARRIEIAAALGLNETQVKIWFQNRRMKQKKRMREIQFEKVNVETCQLGLEG
LT
cgcacaaactttacaaacaaacagttgacggaactggaaaaagaattccattttaacaaatatctcacccgggcc
agaaggatcgagatcgcggcggcgctggggctgaacgagacccaagtgaagatctggttccagaaccggaggatg
aagcagaagaaacgcatgagggagatacagttcgagaaggtcaacgtggagacgtgccagctgggtctcgagggc
ctgact

L. fulica Hox3a:
Primers: L. fulica Hox3a
YLCRPRRIEMAALLNLTERQIKIWFQNRRMKFKKEQRGKNSMDKHGAKSEGSFSGSDTENSSCHGMSGLSGDASP
GIVDCALSKAGTGGGGGNNSGGGAGDNSSGELSGLRLSPNTGQGETNHFLQSRSVRGRSPNTSMGELESACSLQA
QQLRSQGSMQLSHHEQAPLPHQQQQNIKSESPNSGIMSPVQSGVAKLKSHQQQQPPRQPQHQPPSAHTEDNPPTI
PTAPNPYSRGSVHQQRHQPQQHHSGGYGPHPSRHYSASNNMLVTGSMYADINPLDNHTSSHGSMSLHSNINSPVN
CSVSTIGYN
tatctatgccgccccagaagaatagaaatggccgccctgctgaatctgacagagcgacaaataaaaatctggttt
cagaatcgacgaatgaagtttaagaaagagcagcgaggcaagaactccatggacaaacacggagcgaagtccgag
ggcagtttctctggaagcgacacggagaactcctcatgccatggaatgtctggtttaagcggtgatgccagccca
ggaatcgtggactgtgctttatcaaaagccggtacaggaggcggtggagggaacaacagtggaggcggcgcggga
gacaactctagtggagagctcagcggtttacgattatctcccaacacaggccaggggggaaacaaaccacttcctg
cagtcaaggtcagtcaggggtcgctcaccaaacacttccatgggagagctggagtcggcgtgcagcttacaggct
cagcagctccgatcccagggaagcatgcagctgtcgcaccacgagcaagcgcctctgccgcaccaacagcaacag
aacatcaaatccgagagcccgaactctggcatcatgtccccagtacaatccggcgtggccaagctaaaaagccac
cagcaacaacagccacctcgccaaccacagcaccagccaccctctgctcacacagaggacaatcctcctaccata
cctacagcccctaatccttactcccgcgggagcgttcatcaacagcgtcaccaacctcagcaacatcatagcggc
ggttacgggcctcatccgtcaagacactactcagcatctaacaacatgctagtaactggcagtatgtacgcggac
ataaaccccttggacaaccatacgagcagtcacggctcaatgtccctgcactcaaacataaactcaccagtgaat
tgctctgtgtccacaattggctataacca

L. fulica Post1b:
Primers: L. fulica Post1b
REYASSTYISKSRRWELSQLINLSERQIKIWFQNRRIKAKKLQKREDMGMSQNTPNSVLGPSQPPPPSHLM
cgggaatacgcaagctccacctacatctcgaaatcgaggcgatgggaactgtcacagctaattaacctatcagaa
cggcaaatcaaaatctggtttcagaatcgacgaatcaaagccaagaagctacagaagcgggaagacatggggatg
tctcagaacacgcccaacagcgttttgggcccgagtcagcctccgcccccatctcatctcatg

L. immaculata ANTP-B:
Primers: L. fulica ANTPb
RYQTLELEKEFHYNRYLTRRRRIEIAHSLCLTERQIKIWFQ
ttggaaccatatctttatctgcctctccgtcagacacagggagtgggcgatttcgatacgcctgcggcgggttag
gtatcgattgtaatggaattccttttctagctccagggtttggtatct

L. immaculata ANTP:
Primers: Lissachatina ANTP degenerate
RYQTLELEKEFHYNRYLTRRRRIEIAHSLCLTERQIKIW
ccatatctttatctgcctctccgtcagacacagggagtgggcgatttcgatacgcctgcggcgggttaggtatcg
attgtaatggaattccttttctagctccagggtttggtatct

L. immaculate Lox5:
Primers: Lissachatina Lox5 degenerate
RYQTLELEKEFHFNRYLTRRRRIEIAHMLGLTERQIKIWFQNRRMKWKK
cgctaccagaccctagagctggagaaggaattccattttaaccgctacctaactcgccgccgtcgtatagagatt
gcccacatgctggggctgacggaaagacagattaagatttggttccagaaccggagaatgaaatggaagaag

C. hortensis Post2a:
Primers: C. hortensis Post2a
EFLSSSYITRQKRWEISCKLQLSERQVKVWFQNRRMKRKKLTERAKTRIRDDHESKDDEPGGQAQQQPQPHQHSH
PQHLQNEQPHLSQPHQHHQQQQPQHHHHHQPQQHSSQLQHHRSHHNGSGGSSHHHHPHPHHQHHHHIPHQHGSML

PAKPDLA
gagttcctcagtagctcctacatcacccggcagaagcgctgggagatctcctgcaagctgcagctgtccgagcgg
caggtcaaggtttggttccagaaccgacgcatgaagcgaaagaagctcaccgagagggccaagactcgaatccgg
gacgatcatgaaagtaaggacgatgaacctggaggccaggcccagcaacagcctcaacccacaccaacattcacac
cctcagcatcttcaaaatgagcaacctcacttaagtcaaccacatcagcatcaccaacaacaacaaccgcaacat
catcaccatcaccagccacagcagcatagctcacaattacaacaccataggagtcatcacaacggaagtggaggg
agcagccaccaccaccaccctcatcctcaccaccaacatcatcatcatataccacatcaacacgggtctatgcta
ccggccaaaccggaccttgcc

C. aspersum Hox1a:
Primers: C. aspersum Hox1a
Sample 1.1
LTELEKEFHFNKYLTRARRIEIAAALGLNETQVKIWFQNRRMKQKKRMREIQFEKINGESCQLGLEGLTVPN
ttaaccgaactcgagaaggaattccattttaacaaatacctgacgcgggccaggcgtattgaaattgccgctgcc
ctgggtctcaatgaaacacaggtaaaaatctggtttcagaaccggcgaatgaagcagaagaaaagaatgcgggaa
attcagtttgagaagatcaacggagagtcttgtcagctcggcctggaggggctaacggtgccaaat

Sample 1.2
LTELEKEFHFNKYLTRARRIEIAAALGLNETQVKIWFQNRRMKQKKRMREIQFEKINGESCQLGLEGLTVPN
ttaaccgaactcgagaaggaattccattttaacaaatacctgacgcgggccaggcgtattgaaattgccgccgcc
ctgggtctcaatgaaacacaggtaaaaatctggtttcagaaccggcgaatgaagcagaagaaaagaatgcgggaa
attcagtttgagaagatcaacggagagtcttgtcagctcggcctggaggggctaacggtgccaaat

8. Protein model of *Cornu aspersum* Hox1A allele with deletion

Predicted protein models built with AlphaFold 3 for homeodomains of *Cornu aspersum* alleles with (top figure) the supposed 4 amino acid deletion (as in genome reference assembly) and (bottom figure) without the deletion (as in alternative haplotype, all SRA data sets examined and 6 snails tested by PCR) via alphafoldserver.com. With the deletion, helix 2 is shortened and the angle between helices 1 and 3 altered.

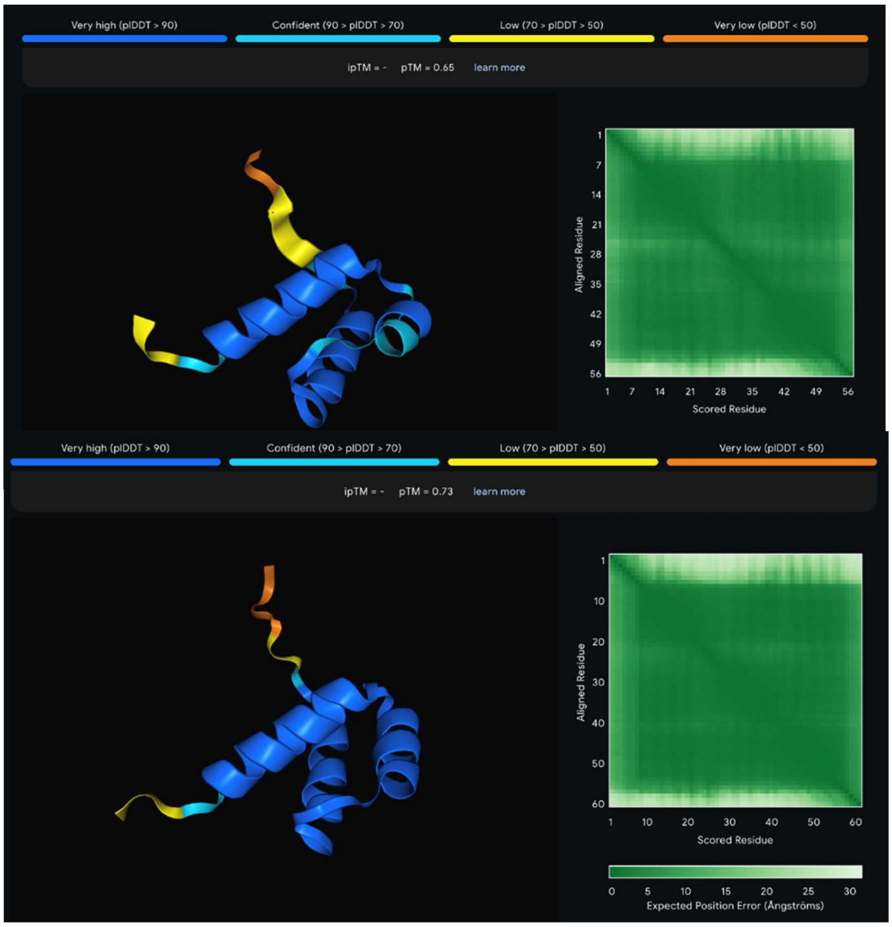

Supplement: Supplementary file 1 — Supporting Material Revision1. [file JEZ-344-363-s001.pdf]
